# Identification of key upper-limb muscles during a standardized reach-to-grasp task toward simplified clinical protocols

**DOI:** 10.3389/fbioe.2026.1786006

**Published:** 2026-04-10

**Authors:** Carmen Cabezaolias, Sergio Garcia, Cristina Sanchez, Rodrigo Rodriguez, Rafael Raya, Eloy Urendes

**Affiliations:** Departamento de Tecnologías de la Información, Escuela Politécnica Superior, Universidad San Pablo-CEU, CEU Universities, Urbanización Montepríncipe, Boadilla del Monte, Spain

**Keywords:** motor control, reach-to-grasp, reproducibility, surface electromyography, upper limb

## Abstract

**Introduction:**

Variability in task execution, target properties, and recording procedures has limited the development of standardized upper-limb surface electromyography (sEMG) reference patterns. Establishing reproducible muscle activation profiles is essential for advancing sEMG-based assessment and motor control applications.

**Methods:**

This study investigated the reproducibility of muscle activation during a highly standardized reach-to-grasp task performed under controlled setup conditions in twenty-seven healthy adults. Participants performed repeated executions of the task under controlled conditions. sEMG signals were processed using time-normalized activation envelopes and cross-correlation analyses allowing for small temporal shifts. Both intra-subject and inter-subject reproducibility were quantified, and a descriptive composite reproducibility measure, combining these two descriptors, was used to summarize overall activation stability.

**Results:**

High reproducibility was observed within subjects, with correlation values ranging from 0.75 to 0.89, and across subjects, with correlations between 0.80 and 0.83. The composite reproducibility analysis highlighted the anterior deltoid, trapezius, lateral deltoid, and biceps brachii as the most reliable contributors to a reproducible activation pattern observed during the reach-to-grasp task.

**Discussion:**

These findings indicate that a reduced subset of muscles can reliably represent the reach-to-grasp movement, supporting the design of simplified electrode configurations that minimize redundancy while preserving essential neuromuscular information. Overall, this work provides a methodological step toward the development of standardized upper-limb activation reference profiles and contributes to the development of efficient sEMG-based assessment, monitoring, and motor control strategies.

## 1 Introduction

The ability to perform Activities of Daily Living (ADLs) is fundamental for maintaining autonomy and an independent lifestyle. Among these activities, the upper limb plays a crucial role, enabling individuals to reach for, grasp, and manipulate objects in their environment. The reach-to-grasp movement is a biomechanically complex upper limb action that unfolds a sequence of coordinated phases: reaching toward an object (target), grasping it, transporting it, and ultimately releasing it (Haggard and Wing, 1995). This apparently simple action requires the precise coordination of multiple joints and muscle groups, from the proximal shoulder and scapular stabilizers to the intrinsic hand muscles. Alterations in this coordinated motor pattern can substantially compromise functional independence, affecting everyday activities such as eating, dressing, or handling tools (Rudolf et al., 2012).

Clinically, alterations in upper-limb function have traditionally been assessed using subjective rating scales or performance-based tests (e.g., Fugl-Meyer, Nine-Hole Peg Test, Box and Block Test) (Santisteban et al., 2016). Although these tools are valuable for identifying gross motor impairments, they lack the sensitivity to detect subtle deficits in inter-muscular coordination or muscle activation patterns (Kalsi-Ryan et al., 2011). Surface electromyography (sEMG) provides an objective approach to quantifying muscle activation, allowing characterization of timing, intensity, and inter-muscular coordination during a task. Its clinical value is well established in gait analysis, where sEMG, often combined with kinematic and kinetic measures, is widely used for functional motor evaluation (Wren et al., 2006; Agostini et al., 2020). A key contributor to the success of gait analysis is the availability of normative reference datasets that enable comparison of patient performance with healthy controls, supporting standardized interpretation and evidence-based clinical decision making (Bovi et al., 2011). However, an equivalent standardized framework for the upper limb is still lacking, limiting the translation of sEMG into routine functional assessment. Establishing reproducible reference activation patterns for functional upper-limb tasks would therefore represent an important step toward enabling more quantitative and standardized sEMG-based assessments, supporting the development of objective evaluation frameworks similar to those successfully implemented in gait analysis (Jarque-Bou et al., 2021).

Despite several exploratory studies investigating upper-limb sEMG during functional tasks, the literature remains highly heterogeneous due to inconsistencies in protocols and analysis approaches. In motor control and neurorehabilitation research, analytical paradigms such as the center-out reaching task have been widely used because they provide highly controlled kinematic conditions and well-characterized muscle activation patterns (Úbeda et al., 2017). However, these paradigms typically involve point-to-target movements without object manipulation and therefore they do not capture the functional reach-to-grasp actions commonly performed in activities of daily living. Moreover, muscle activation during reach-to-grasp varies with object characteristics and spatial configuration, which has hindered the establishment of a normative activation pattern (Bonnefoy et al., 2009; Fligge et al., 2013). This lack of standardization limits cross-study comparability and hinders clinical interpretation. A controlled and standardized approach is therefore needed to isolate the core activation pattern of this movement and develop reproducible reference activation patterns. To the best of our knowledge, no previous study has combined strict task standardization with a systematic analysis of upper-limb muscle activation to identify the most reproducible muscle set required for establishing reproducible activation references.

The reach-to-grasp movement is well suited for standardization due to its functional relevance, reproducibility, and biomechanical consistency. A well-defined reference task is needed to enable reliable comparisons and support future methodological developments. In this study, we employed a simple and highly reproducible task: reaching for and drinking from an empty cup. This task captures the main phases of reaching, grasping, lifting, and releasing, while allowing precise and repeatable sEMG acquisition. This controlled setup improves internal validity and provides a stable platform for systematically testing the influence of object or task parameters. Furthermore, identifying the muscles that consistently contribute to this task may support the definition of a reduced yet informative sEMG channel configuration, improving feasibility in clinical and research environments.

This study aimed to characterize upper-limb muscle activation during a standardized reach-to-grasp task in healthy adults and assess the reproducibility of the resulting sEMG activation patterns. We also aimed to identify a reduced subset of muscles that reliably represent this functional movement, allowing the number of sEMG channels to be reduced without loss of task-related information. Establishing such evidence may support the development of optimized and standardized sEMG acquisition protocols for upper-limb assessment, facilitating their implementation in clinical and research environments.

## 2 Materials and methods

### 2.1 Participants

27 healthy participants (13 males, 14 females; mean ± SD age: 20.9 ± 2.0 years), all right-handed, participated in this study. None reported neurological or musculoskeletal disorders affecting upper-limb function. All participants provided written informed consent prior to participation. The study was approved by the Research Ethics Committee of CEU San Pablo University, Madrid, Spain (reference number: 561/21/53).

### 2.2 Experimental setup

**FIGURE 1:**
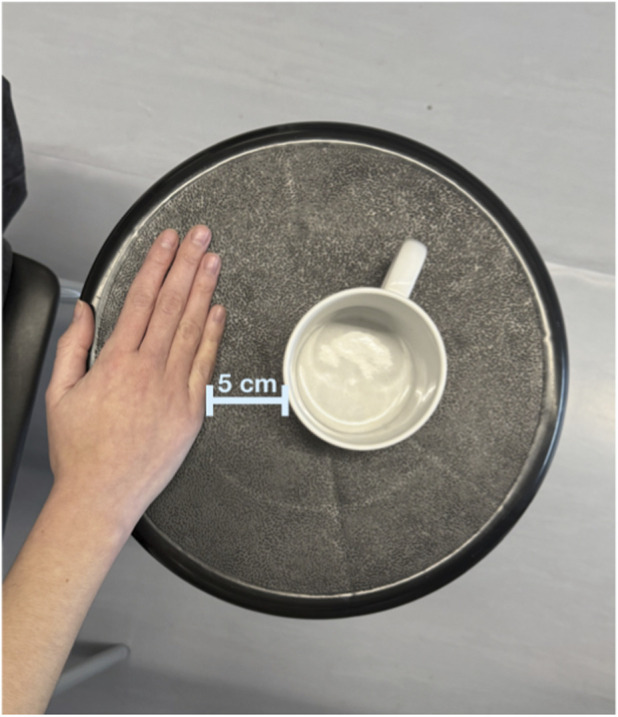
Initial posture of the participant and cup position for the standardized reach-to-grasp task.

Participants were seated with their back supported and feet flat on the floor to standardize posture. The dominant (right) upper limb was positioned with the elbow flexed at 90° and the forearm in a neutral position, resting on an adjustable-height support placed in front of the participant. A standard ceramic cup (8 cm diameter, 330 g empty mass) was placed at a fixed distance of 5 cm in front of the participant’s resting hand position (Figure 1). The object was selected because grasping a cup represents a simple and reproducible functional task commonly encountered in daily activities. Participants were instructed to grasp the cup by its body to ensure a consistent grasp configuration across subjects. This setup ensured a uniform starting position and minimized variability in task execution.

### 2.3 Data acquisition and preprocessing

**FIGURE 2:**
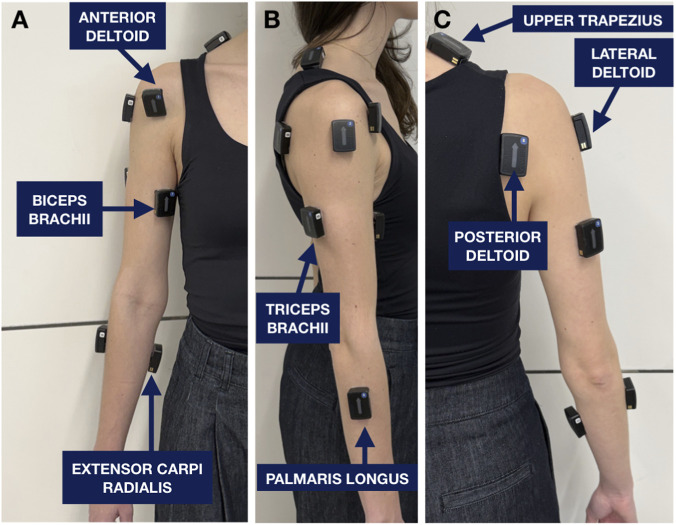
Placement of Delsys Trigno Avanti sEMG sensors on upper-limb following SENIAM recommendations. **(A)** Anterior view showing electrodes over the anterior deltoid, biceps brachii, and extensor carpi radialis. **(B)** Lateral view showing electrodes on the triceps brachii and palmaris longus. **(C)** Posterior view showing electrodes over the upper trapezius, lateral deltoid, and posterior deltoid.

sEMG signals were recorded using a Delsys Trigno Avanti wireless system (Delsys Inc., Boston, MA, USA). Eight bipolar Ag/AgCl electrodes were placed over the dominant upper-limb muscles according to SENIAM guidelines to ensure reproducible placement and high-quality signal acquisition (Figure 2) (Hermens et al., 2000). The monitored muscles were the biceps brachii, triceps brachii, anterior deltoid, lateral deltoid, posterior deltoid, upper trapezius, palmaris longus, and extensor carpi radialis. These muscles were selected to represent the main functional contributions of the shoulder, elbow, and wrist during reaching and object manipulation, while remaining suitable for reliable surface EMG recording.

Signals were sampled at 2148 Hz with 16-bit resolution and a common-mode rejection ratio greater than 80 dB. Raw sEMG data were stored for offline processing in MATLAB (R2024b, MathWorks, Natick, MA, USA).

**FIGURE 3:**
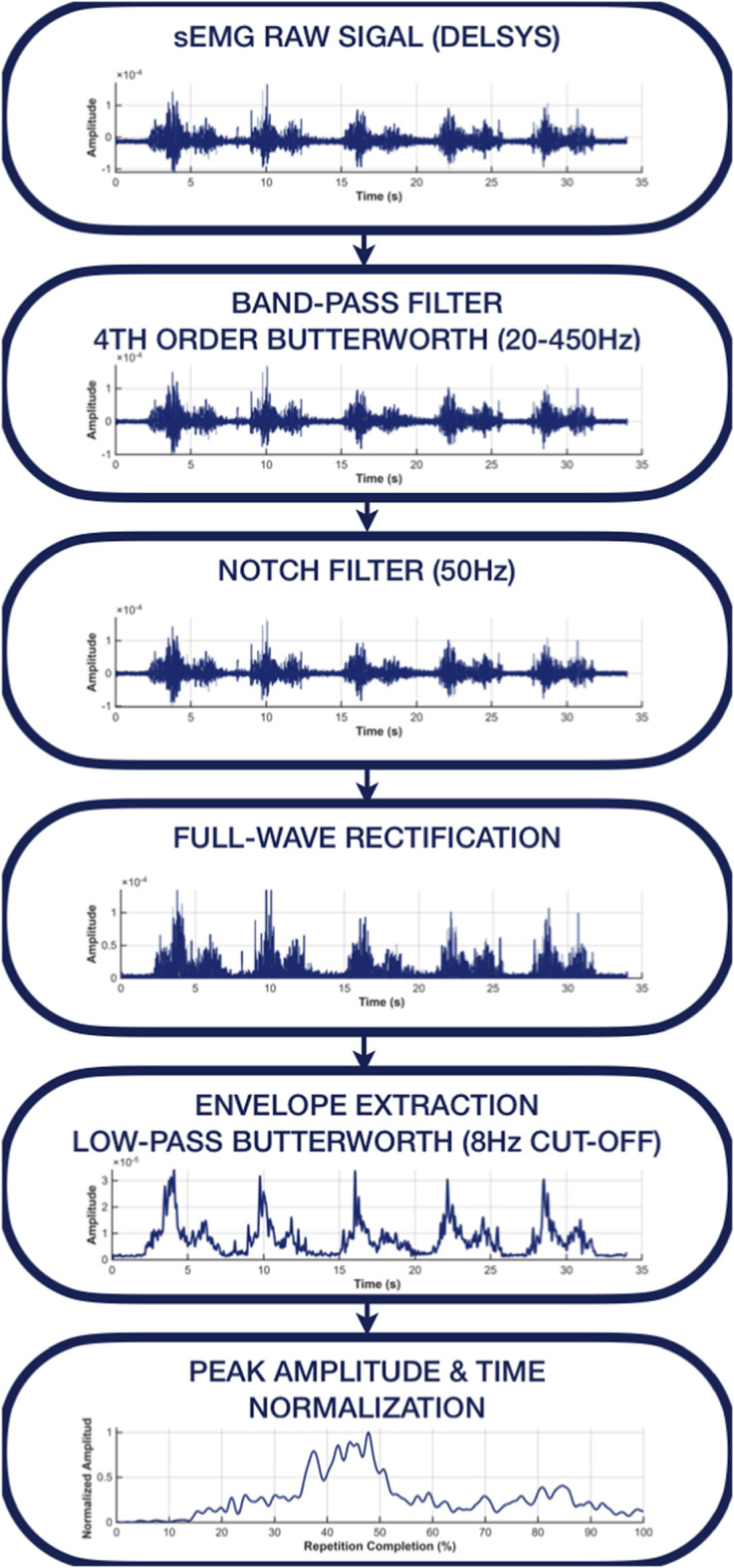
sEMG signal preprocessing pipeline. Raw signals recorded with the Delsys system were band-pass filtered (20–450 Hz, fourth-order Butterworth) and processed with a 50 Hz notch filter to remove power-line interference. Signals were then full-wave rectified and low-pass filtered (8 Hz Butterworth) to obtain the linear envelope. Finally, envelopes were peak-amplitude normalized and time-normalized to 100% of the movement cycle prior to repeatability and reproducibility analyses.

To enhance signal quality and enable meaningful intra- and inter-subject comparisons, all sEMG signals were processed using a standardized preprocessing pipeline (Figure 3). This pipeline was designed to preserve the physiological characteristics of the signal while reducing noise and variability unrelated to task execution. The following steps were applied:

First, signals were band-pass filtered using a fourth-order zero-lag Butterworth filter (20–450 Hz) to attenuate low-frequency motion artefacts and high-frequency noise while preserving the physiological sEMG spectrum. A 50 Hz notch filter was then applied to suppress power-line interference (European AC standard) without distorting the underlying muscle activation signal.

Filtered signals were full-wave rectified to convert the bipolar waveform into a unipolar signal suitable for amplitude-based analysis (Albert and Shadmehr, 2016). Subsequently, a linear envelope was extracted using a fourth-order zero-lag low-pass Butterworth filter with an 8 Hz cutoff, providing a smooth representation of the overall muscle activation profile while preserving its temporal structure.

For each subject and muscle, sEMG envelopes were amplitude-normalized to the maximum activation value observed across the five task repetitions for that specific muscle in that participant. This peak value served as a subject- and muscle-specific scaling factor, so that all repetitions were expressed relative to the maximum activation observed during the task. Because the task execution was highly standardized and signals were inspected during preprocessing, no abnormal peaks were observed that could disproportionately influence the normalization. This peak-based normalization reduced inter-subject amplitude variability while preserving relative activation patterns and was selected over maximum voluntary contraction (MVC) normalization due to its greater feasibility and applicability across diverse populations, including individuals with motor impairments (Burden, 2010; Sarcher et al., 2017; Besomi et al., 2020).

Finally, all normalized sEMG envelopes were time-normalized to 100% of the movement cycle using linear interpolation, allowing temporal alignment and direct comparison across repetitions and subjects.

### 2.4 Experimental procedure

**FIGURE 4:**
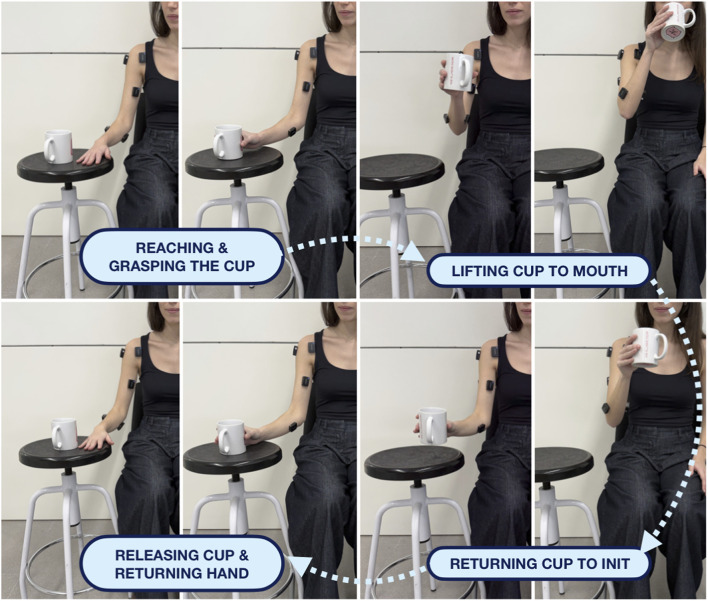
Standardized reach-to-grasp task. Each trial consisted of four sequential phases replicating a drinking gesture: (1) reaching and grasping the cup, (2) lifting the cup towards the mouth, (3) returning the cup to the initial position, and (4) releasing the cup and returning the hand to the starting posture. Five repetitions were performed per participant under standardized posture and task conditions.

Participants performed a standardized reach-to-grasp task replicating a drinking gesture. Each trial comprised four sequential phases: (1) reaching and grasping the cup, (2) lifting the cup towards the mouth, (3) returning the cup to the initial position, and (4) releasing the cup and returning the hand to the starting posture (see Figure 4). Five repetitions were recorded per participant. Movements were self-paced to preserve natural variability in movement duration, while task conditions such as posture, object characteristics, target distance, and grasp configuration were strictly standardized. A previous familiarization trial was performed prior to data collection to ensure task understanding and consistent execution.

### 2.5 Data analysis

Data analysis aimed to quantify the repeatability (within-subject consistency across repetitions) and reproducibility (between-subject similarity across participants) of muscle activation patterns and to identify the muscles that most consistently contributed to the reach-to-grasp movement. All analyses were performed using MATLAB (R2024b, MathWorks, Natick, MA, USA).

For each subject and muscle, time-normalized sEMG envelopes from the five repetitions were first averaged to obtain a representative activation profile. This averaging step reduced trial-to-trial variability and provided a single subject-level activation profile for subsequent analyses.

Intra-subject repeatability was assessed by computing the cross-correlation between each pair of repetitions for the same muscle using the time-normalized linear envelopes of the sEMG signals, representing muscle activation amplitude across the normalized movement cycle. To prevent small trial-to-trial temporal shifts in activation peak timing from artificially reducing repeatability values, cross-correlation was computed allowing a temporal lag of ±25 samples. Because the signals were time-normalized to 100 points representing the full movement cycle (0%–100%), this lag corresponds to a shift of ±25% of the cycle. This approach is consistent with previous studies using cross-correlation to compare dynamic sEMG patterns, in which limited temporal shifts are permitted to account for natural neuromuscular timing variability while preserving the overall activation structure (Wren et al., 2006; Soares et al., 2013).

The maximum cross-correlation coefficient within this lag window was taken as the repeatability metric, ensuring that within-subject repeatability reflected similarity in activation pattern shape and overall timing structure rather than sensitivity to minor physiological timing offsets. The mean coefficient per muscle was used as an indicator of within-subject repeatability of activation timing and profile shape (Wren et al., 2006).

Inter-subject reproducibility was evaluated by computing the cross-correlation between the mean activation profiles of each muscle across participants using the same lag allowance. This metric quantified the extent to which a common activation pattern was shared across individuals. Cross-correlation was selected because it preserves the physiological temporal structure of activation while still tolerating small phase shifts, which is essential in dynamic motor tasks.

For descriptive analysis, the mean and standard deviation of intra- and inter-subject cross-correlation values were calculated for each muscle. Group-level mean activation profiles with standard deviation were also derived for each muscle to illustrate overall activation patterns across participants. Additionally, a descriptive composite reproducibility score was calculated for each muscle as the arithmetic mean of its intra- and inter-subject cross-correlation coefficients. This composite measure was defined as the arithmetic mean of the intra- and inter-subject coefficients and was introduced as a descriptive summary of overall activation reproducibility. It was used to facilitate the identification of the most informative muscles for a reduced-channel sEMG configuration, rather than as a validated or standardized index.

## 3 Results

The preprocessing pipeline generated clean and smooth activation profiles, suitable for intra- and inter-subject comparisons.

### 3.1 Intra-subject repeatability of muscle activation patterns

Intra-subject cross-correlation analysis revealed a high degree of repeatability across repetitions for most muscles. Table 1 presents the mean cross-correlation coefficients and their standard deviations, averaged across repetition pairs and subjects, for each muscle. The correlation values ranged from 0.75 (Palmaris Longus) to 0.89 (Anterior Deltoid), indicating overall stable activation patterns across repeated trials. Muscles such as the anterior deltoid (0.89 ± 0.09) and trapezius (0.87 ± 0.04) showed the highest repeatability, whereas slightly lower values were observed for the palmaris longus and extensor carpi radialis, indicating greater variability between repetitions.

**TABLE 1:** Intra-subject cross-correlation (mean ± SD) per muscle.

Cross-correlation	Muscle							
	BB	TB	AD	T	LD	PD	PL	ECR
Mean ± SD	0.85 ± 0.06	0.81 ± 0.07	0.89 ± 0.09	0.87 ± 0.04	0.86 ± 0.03	0.81 ± 0.06	0.75 ± 0.08	0.78 ± 0.09

Values are mean ± SD, over 8 muscles: biceps brachii (BB), triceps brachii (TB), anterior deltoid (AD), trapezius (T), lateral deltoid (LD), posterior deltoid (PD), palmaris longus (PL), extensor carpi radialis (ECR).

### 3.2 Inter-subject reproducibility of activation patterns

Inter-subject cross-correlation analysis revealed a high degree of reproducibility among participants for most muscles. Table 2 presents the average inter-subject cross-correlation coefficients and their standard deviation for each of the eight evaluated muscles. The correlation values ranged between 0.80 (Palmaris Longus) and 0.83 (Triceps Brachii).

**TABLE 2:** Inter-subject cross-correlation (mean ± SD) per muscle.

Cross-correlation	Muscle							
	BB	TB	AD	T	LD	PD	PL	ECR
Mean ± SD	0.80 ± 0.05	0.83 ± 0.04	0.81 ± 0.05	0.81 ± 0.05	0.80 ± 0.06	0.82 ± 0.04	0.80 ± 0.06	0.80 ± 0.05

Values are mean ± SD, over 8 muscles: biceps brachii (BB), triceps brachii (TB), anterior deltoid (AD), trapezius (T), lateral deltoid (LD), posterior deltoid (PD), palmaris longus (PL), extensor carpi radialis (ECR).

**FIGURE 5:**
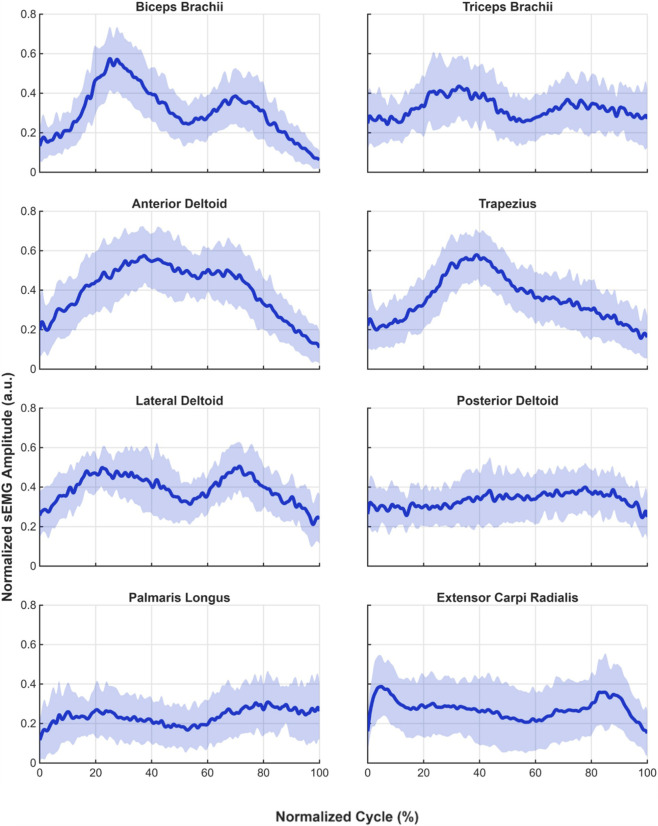
Normalized mean sEMG activation profiles for each muscle across the full movement cycle for all subjects. Solid lines represent the mean activation curve across participants, while shaded areas indicate the standard deviation.

Figure 5 shows the normalized mean sEMG activation curves for each muscle throughout the full movement cycle for all subjects. The solid lines represent the mean activation profile across participants, while the shaded areas indicate the corresponding standard deviation. These profiles were obtained from sEMG signals normalized to their maximum activation value and time-normalized to the movement cycle.

### 3.3 Identification of the most reproducible muscles

**FIGURE 6:**
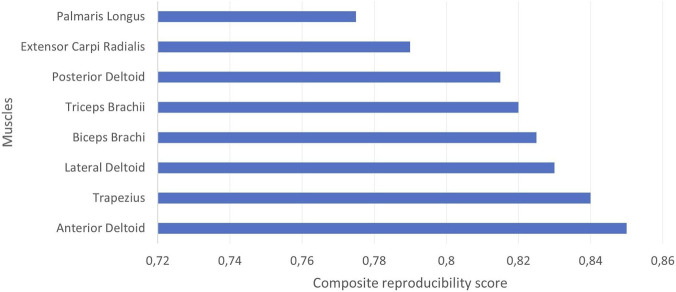
Descriptive ranking of muscles based on their composite reproducibility score during the reach-to-grasp task. The score corresponds to the arithmetic mean of the intra- and inter-subject cross-correlation coefficients for each muscle.

Figure 6 shows a descriptive ranking of muscles based on their overall sEMG activation reproducibility during the reach-to-grasp task, quantified using a composite score derived from intra- and inter-subject cross-correlation coefficients. The highest values were observed for the anterior deltoid (0.85), trapezius (0.84), lateral deltoid (0.83), and biceps brachii (0.83). Intermediate values were found for the triceps brachii (0.82) and posterior deltoid (0.81), while lower scores were obtained for the extensor carpi radialis (0.79) and palmaris longus (0.77). Overall, the composite scores ranged from 0.77 to 0.85 across the evaluated muscles.

## 4 Discussion

This study shows that a standardized reach-to-grasp task elicits highly reproducible intra-subject sEMG activation, with proximal muscles displaying the greatest stability across repeated executions. The anterior deltoid, trapezius, lateral deltoid and biceps brachii presented the highest intra-subject correlations (0.89 ± 0.09, 0.87 ± 0.04, 0.86 ± 0.03 and 0.85 ± 0.06, respectively), indicating that these muscles are well suited to represent the most stable and consistently recruited components of the activation structure associated with the reach-to-grasp task under the proposed standardized conditions, particularly in reduced-channel sEMG configurations. In contrast, distal muscles such as the palmaris longus and extensor carpi radialis showed lower intra-subject repeatability (0.75 ± 0.08 and 0.78 ± 0.09), which aligns with the greater inherent variability of wrist–hand control. This difference may also be influenced by the smaller size and closer anatomical arrangement of distal muscles, which can increase variability in sEMG recordings. Overall, these findings suggest that proximal muscles provide more reliable within-subject signal sources for applications such as classification or intent detection, whereas the inclusion of distal muscles requires stricter task control to ensure stable and interpretable activation patterns.

Previous studies have examined how factors such as object properties or reaching distance influence upper-limb muscle activation, but these works primarily focused on task-dependent modulation rather than on reproducibility under strictly standardized conditions. For example, Bonnefoy et al. (Bonnefoy et al., 2009) reported increased proximal activation with greater reaching distances, and Fligge et al. (Fligge et al., 2013) showed that forearm sEMG can discriminate object size or weight, yet both focused on task-dependent modulation rather than identifying highly reproducible activation profiles obtained under controlled and repeatable conditions. In this context, the present study addresses this gap by providing a reproducible benchmark task that isolates the underlying activation structure, offering a degree of standardization that previous works did not establish.

Inter-subject reproducibility was also high, indicating that healthy adults share a consistent neuromuscular activation pattern when the task is performed under the same controlled conditions. Correlation values were consistently close to 0.80 across all muscles, with triceps brachii (0.83 ± 0.04), posterior deltoid (0.82 ± 0.04), and anterior deltoid and trapezius (0.81 ± 0.05, both) showing the strongest between-subject alignment. The use of cross-correlation with lag compensation and time-normalized activation profiles was crucial for isolating the underlying activation structure, ensuring that physiological activation pattern similarity was captured even in the presence of natural timing variability across individuals. These results support the use of the proposed methodology for identifying reproducible reference activation patterns and highlight its potential relevance for future signal-based assessment and machine learning applications.

However, the present analysis considered the entire movement cycle as a single unit. Although the reach-to-grasp action includes biomechanically distinct phases (reaching, lifting, returning, and release), the objective of this study was to evaluate the global reproducibility of the activation pattern under standardized conditions. Phase-specific analyses could reveal phase-dependent variations in muscle activation stability that may not be fully captured by a global cross-correlation approach. Future studies incorporating phase-segmented analyses could therefore provide additional insight into the neuromuscular control of different stages of the reach-to-grasp movement, particularly in clinical populations. One of the most relevant findings is the identification of a reduced subset of muscles that consistently exhibited the highest reproducibility across subjects and repetitions. The anterior deltoid, trapezius, lateral deltoid, and biceps brachii captured the most reproducible and informative features of the reach-to-grasp movement, with minimal redundancy. This contributes directly to one of the practical goals stated at the end of the introduction: defining an informative yet minimal sEMG configuration that reduces acquisition complexity without compromising task-related information. Such reduced-channel setups are highly valuable for developing wearable systems, simplifying clinical protocols, and improving computational efficiency in control and classification pipelines. While inter-muscular coordination metrics such as co-contraction indices can provide additional insight into motor control, their interpretation in functional multi-joint tasks such as reach-to-grasp may be influenced by task-dependent biomechanical adaptations; therefore, the present analysis focused on identifying muscles that exhibit stable and reproducible individual activation profiles.

Beyond the specific task analyzed, these findings may also be relevant for rehabilitation of neuromotor disorders, where the absence of standardized upper-limb assessments limits objective evaluation. The high reproducibility observed suggests that a simple reach-to-grasp task can serve as a reliable methodological reference for tracking individual progress and identifying deviations from reproducible activation patterns. Moreover, the identification of a reduced set of highly reproducible muscles supports the development of simplified electrode configurations, facilitating the use of sEMG-based tools and pattern recognition models in clinical settings. In this context, standardized reach-to-grasp paradigms may contribute to the development of objective upper-limb assessment frameworks, analogous to the role that standardized gait analysis has played in lower-limb rehabilitation.

Several limitations should be acknowledged. The study was conducted with young healthy adults, and extending the analysis to clinical populations will be essential to determine how pathological motor patterns differ from the reproducible activation patterns identified in this study. In addition, the strict standardization that enabled the high reproducibility observed also limited the examination of how variations in object characteristics or movement demands influence muscle activation. Future work integrating sEMG with kinematic or kinetic measurements may provide deeper insight into neuromuscular coordination and support the development of multimodal assessment or control frameworks.

## 5 Conclusion

The results demonstrate that the proposed standardized reach-to-grasp task, together with a rigorously structured sEMG processing workflow, yields reproducible activation profiles across healthy individuals. This consistency supports the use of the protocol as a methodological basis for future upper-limb sEMG reference datasets and for identifying reduced yet informative channel configurations. By reducing acquisition complexity and preserving the main features of muscle activation patterns, the proposed approach enables more practical and repeatable sEMG-based functional assessment protocols for clinical and rehabilitation applications.

## Data Availability

The datasets generated for this study can be found in the Zenodo repository at https://doi.org/10.5281/zenodo.17950086. The processed datasets are available at https://doi.org/10.5281/zenodo.17950223. The MATLAB (R2024b, MathWorks, Natick, MA, USA) code used for data processing and analysis is publicly available in Zenodo at https://doi.org/10.5281/zenodo.17951424.
